# Supplementary material for: Vitamin D Status for Chinese Children and Adolescents in CNNHS 2016–2017
Source: Nutrients. 2022 Nov 21;14(22):4928. doi: 10.3390/nu14224928 (PMC9693967; doi:10.3390/nu14224928)
Supplement: Supplementary file 1 [file nutrients-14-04928-s001.zip › nutrients-2028144-supplementary.pdf]

Table S1. Odds Ratio of abdominal obesity on vitamin D insufficiency and deficiency (OR, 95%CI)

Status	Total	Boy	Girl
Vitamin D insufficiency	1.11 (1.02–1.21)<br>*P* = 0.018	1.11 (0.984–1.26)<br>*P* = 0.088	1.115 (0.99–1.26)<br>*P* = 0.080
Vitamin D deficiency	1.25 (1.11–1.42)<br>*P* = 0.004	1.32(1.09–1.60)<br>*P* = 0.005	1.211(1.03–1.43)<br>*P* = 0.021
